# Sociodemographics and Transdiagnostic Mental Health Symptoms in SOCIAL (Studies of Online Cohorts for Internalizing Symptoms and Language) I and II: Cross-sectional Survey and Botometer Analysis

**DOI:** 10.2196/39324

**Published:** 2022-10-20

**Authors:** Lorenzo Lorenzo-Luaces, Jacqueline Howard, Andy Edinger, Harry Yaojun Yan, Lauren A Rutter, Danny Valdez, Johan Bollen

**Affiliations:** 1 Department of Psychological and Brain Sciences Indiana University-Bloomington Bloomington, IN United States; 2 Luddy School of Informatics, Computing, and Engineering Indiana University-Bloomington Bloomington, IN United States; 3 Media School Indiana University-Bloomington Bloomington, IN United States; 4 School of Public Health Indiana University-Bloomington Bloomington, IN United States; 5 Cognitive Science Program Indiana University-Bloomington Bloomington, IN United States

**Keywords:** depression, anxiety, pain, alcohol, social media

## Abstract

**Background:**

Internalizing, externalizing, and somatoform disorders are the most common and disabling forms of psychopathology. Our understanding of these clinical problems is limited by a reliance on self-report along with research using small samples. Social media has emerged as an exciting channel for collecting a large sample of longitudinal data from individuals to study psychopathology.

**Objective:**

This study reported the results of 2 large ongoing studies in which we collected data from Twitter and self-reported clinical screening scales, the Studies of Online Cohorts for Internalizing Symptoms and Language (SOCIAL) I and II.

**Methods:**

The participants were a sample of Twitter-using adults (SOCIAL I: N=1123) targeted to be nationally representative in terms of age, sex assigned at birth, race, and ethnicity, as well as a sample of college students in the Midwest (SOCIAL II: N=1988), of which 61.78% (1228/1988) were Twitter users. For all participants who were Twitter users, we asked for access to their Twitter handle, which we analyzed using Botometer, which rates the likelihood of an account belonging to a bot. We divided participants into 4 groups: Twitter users who did not give us their handle or gave us invalid handles (*invalid*), those who denied being Twitter users (n*o Twitter,* only available for SOCIAL II), Twitter users who gave their handles but whose accounts had high bot scores (*bot-like*), and Twitter users who provided their handles and had low bot scores (*valid*). We explored whether there were significant differences among these groups in terms of their sociodemographic features, clinical symptoms, and aspects of social media use (ie, platforms used and time).

**Results:**

In SOCIAL I, most individuals were classified as valid (580/1123, 51.65%), and a few were deemed bot-like (190/1123, 16.91%). A total of 31.43% (353/1123) gave no handle or gave an invalid handle (eg, entered “N/A”). In SOCIAL II, many individuals were not Twitter users (760/1988, 38.23%). Of the Twitter users in SOCIAL II (1228/1988, 61.78%), most were classified as either invalid (515/1228, 41.94%) or valid (484/1228, 39.41%), with a smaller fraction deemed bot-like (229/1228, 18.65%). Participants reported high rates of mental health diagnoses as well as high levels of symptoms, especially in SOCIAL II. In general, the differences between individuals who provided or did not provide their social media handles were small and not statistically significant.

**Conclusions:**

Triangulating passively acquired social media data and self-reported questionnaires offers new possibilities for large-scale assessment and evaluation of vulnerability to mental disorders. The propensity of participants to share social media handles is likely not a source of sample bias in subsequent social media analytics.

## Introduction

### Background

So-called mental disorders, including depression, anxiety, substance use, and pain-related conditions, account for a substantial proportion of disabilities attributed to illness worldwide [1]. According to hierarchical models of psychopathology [2], most of these clinical problems can be grouped into dimensions that include an *internalizing* dimension, involving emotional dysfunction, and an *externalizing* dimension, involving disinhibition or antagonism. Research implicates various mechanisms in the etiology and maintenance of mental disorder symptoms, including sustained negative affect, disturbances in positive affect, disrupted social processes, disturbances in arousal and regulatory processes, sensorimotor problems, and cognitive dysfunction [3]. However, it has been extremely difficult to determine reliable mechanisms of psychopathology. Although mental disorders are very common [4], they are also highly heterogeneous in their presenting characteristics [2]. In addition, the longitudinal course of mental health symptoms is also heterogeneous, with some individuals having brief courses and others having highly chronic or relapsing-recovering courses [5].

### Social Media

Characterizing heterogeneity in psychopathology requires large samples, which, as a result, have become a staple of modern clinical research, that is, clinical trials such as STAR*D (Sequenced Treatment Alternatives to Relieve Depression) [6], epidemiological studies [7], neuroimaging cohorts [8], and neurocognitive assessment studies [9]. More recently, analyses of naturalistic social media samples have also facilitated the collection of large samples. Social media is well-suited for collecting research data because it is ubiquitous in modern life; 72% of adults in the United States report belonging to at least one social media platform [10]. Twitter, specifically, is used by 23% of the population in the United States [10]. Although the use of Twitter has a Pareto distribution, wherein a few individuals account for most of the active Twitter activity; approximately three-fourths of Twitter users use the platform at least once a week (46% use it daily and 27% use it at least weekly). As a social media platform, Twitter is geared toward sharing frequent, brief, and introspective posts that are suitable for longitudinal, within-subject text analysis at high temporal resolutions.

We had used Twitter previously to study vulnerability to mental health symptoms. For example, in a study, we reported that individuals who had disclosed that they were diagnosed with depression in their tweets (eg, “I was diagnosed with depression a couple of months ago...”) had different circadian patterns of Twitter activity than a random sample of Twitter users [11]. Specifically, individuals who disclosed a depression diagnosis used Twitter more frequently later into the night and used Twitter less frequently earlier in the day, possibly indicating circadian differences between the depressed users and the random sample. In another study, we measured lexical proxies of cognitive distortions, words like “should,” “must,” “have to,” “nobody,” or “always,” a concept from the literature on cognitive behavioral therapy which points to rigid or inflexible thinking [12,13]. As suggested by the generic cognitive model underlying cognitive behavioral therapy [14], individuals with depression make more use of cognitive distortions than a random sample of individuals [15]. Al-Mosaiwi and Johnstone [16] reported a similar finding with language that they deemed “absolutist.” Others have also found associations between features of written text and depressive symptoms. For example, greater use of personal pronouns (eg, “I”) in social media and other contexts appears to be correlated to symptoms of depression [17], a finding that connects with research using cognitive tasks linking depression to increased self-referential processing [18]. Similarly, greater use of negative emotional words, including those expressing depressive symptoms, appears to be related to depressive symptoms [19].

In spite of the potential offered by social media data for research into the mechanisms involved in the development and maintenance of mental disorders, there are limitations to passively acquired social media data. A limitation is that social media users are not representative of the general population [10,20]. There are data on sociodemographic differences between individuals who use specific social media sites and those who do not. Relative to the broader population, Twitter users are more likely to be male, younger, more educated, and more liberal leaning in their political orientation [10].

It has also been hypothesized that differences in variables such as need for self-disclosure [20] may bias samples of individuals who are on Twitter versus those who are not. Likewise, individuals who volunteer to give researchers access to their social media accounts may provide a biased subsample of individuals with a stronger disposition to self-disclose. Another limitation to using social media data for research is that researchers lack information to support inferences about participants’ health from their web-based activity (eg, Is someone actually depressed even if they explicitly said so?).

### This Study

To address these limitations of social media research, namely the lack of sample representativeness and inability to verify health status, we conducted the Studies of Online Cohorts for Internalizing Symptoms and Language (SOCIAL). SOCIAL are cohort-based studies in which we triangulated self-reported disorder screening questionnaires with data acquired from social media. Participants in SOCIAL I and II completed a series of disorder screening questionnaires focused on internalizing symptoms that are meant to capture psychopathology more broadly. They were also asked to provide their Twitter handles which we subsequently verified for validity, including how closely they resembled the behavior of bots. SOCIAL I is a sample of Twitter users (Methods section) targeted to be nationally representative in terms of age, sex assigned at birth, race, and ethnicity, and SOCIAL II is a large sample of college students.

Here, we describe the baseline sociodemographic characteristics, social media use data, and mental health characteristics of individuals in SOCIAL I and II. Because we asked individuals to self-report whether they used Twitter and to give us access to their Twitter accounts, we could compare sociodemographic characteristics, social media use data, and mental health differences between groups of individuals depending on their willingness to share their social media data. We distinguished approximately 2 groups of participants: those who provided *valid* Twitter handles pointing to their own social media content and those who did not or refused. The latter group can be separated into three subgroups: (1) users who refused to provide a valid Twitter handle (*invalid handle*), (2) users who denied being Twitter users (*not a Twitter user*), and (3) users who did provide an existing Twitter handle, but the accounts were deemed to be *bot-like* as defined by a machine learning classifier [21].

## Methods

### Overview

Both SOCIAL samples answered self-reported questionnaires probing internalizing, externalizing, somatoform, and thought disorder symptoms (Table 1). We also collected demographic information and aspects of social media use, including whether the individual was a Twitter user, whether they were willing to let us access their Twitter time line, and which other social media platforms they used.

**Table 1:** Assessment of psychopathology for the Studies of Online Cohorts for Internalizing Symptoms and Language (SOCIAL I, N=1123 and SOCIAL II, N=1988).

Construct			Measure		Item		Response options		Original range		Cronbach α
**Internalizing**											
	Depression	PHQ^a^-9		9		0-3 (not at all to nearly every day)		0-27		.90	
	Stress	MIDUS^b^		9		0-10 (no stress to severe stress)		0-90		.84	
	Social anxiety	DSM severity^c^		10		0-4 (never to all of the time)		0-40		.94	
	Panic	DSM severity		10		0-4 (never to all of the time)		0-40		.95	
	Agoraphobia	DSM severity		10		0-4 (never to all of the time)		0-40		.96	
	Worry	DSM severity		10		0-4 (never to all of the time)		0-40		.93	
**Somatoform**											
	Pain	PHQ-15		15		0-2 (not bothered a lot to bothered a lot)		0-30		.86	
	Insomnia	ISI^d^		7		Varies with each question		0-28		.87	
**Externalizing**											
	Alcohol use	AUDIT^e^		10		Varies with each question		0-40		.90	
	Substance use	DSM severity		10		0-4 (not at all to nearly every day)		0-40		.89	
**Thought disorder**											
	Hypomania	ASRM^f^		5		Varies with each question		0-25		.82	

^a^PHQ: Patient Health Questionnaire.

^b^MIDUS: Midlife in the United States self-reported measure of perceived stress.

^c^DSM severity: Diagnostic and Statistical Manual of Mental Disorders severity measure for each symptom.

^d^ISI: Insomnia Severity Index.

^e^AUDIT: Alcohol Use Disorders Identification Test.

^f^ASRM: Altman Self-Rating Mania.

### Participant Recruitment

SOCIAL I purposefully sampled individuals via Qualtrics panels. We aimed to recruit approximately 1000 Twitter users, given the budgetary constraints for this study. Individuals were recruited from July 2020 to March 2021 for a study on “social media and mental health.” The sample was selected to represent the United States at the intersections of age, gender, race and ethnicity.

All individuals in SOCIAL I were Twitter users. Accordingly, we could not ascertain the role that being a Twitter user in itself has on potential differences between individuals in baseline sociodemographic characteristics, social media use, and mental health symptoms. To have a sample of individuals who did not use Twitter as well as to have an additional sample with which to assess the transportability of results from SOCIAL I, we began the SOCIAL II study. SOCIAL II recruited college students from a predominantly White and Asian university in the Midwest. Individuals were compensated with credits in an introductory psychology course. Individuals were recruited from September 2020 to the present date.

### Measures

For individuals in SOCIAL I and SOCIAL II, we collected information on characteristics described in the following sections.

#### Demographic Characteristics

Specifically, we collected age, political orientation on a 10-point Likert scale (1=extremely liberal, 10=extremely conservative), race, ethnicity, sex assigned at birth (male, female, other or inconclusive, or prefer not to say), gender identity (male, female, nonbinary, genderqueer, agender, other, or prefer not to say), and sexual orientation (heterosexual or straight, homosexual or gay, bisexual or pansexual, other, or prefer not to say). In SOCIAL I, we asked participants for their annual household income. In SOCIAL II, we asked participants to estimate their *parents’* annual household income. We present both of these as the same variable (ie, estimated household income). In addition, in SOCIAL I, we asked participants to indicate their race by using a single category from a list (White, Black or African American, American Indian or Alaska Native, Native Hawaiian or Pacific Islander, Hispanic, or other). In SOCIAL II, we allowed participants to select multiple racial and ethnic identities, including all the possibilities in SOCIAL I along with Middle Eastern or North African. We recoded the categories in SOCIAL II to fit a version of the race variable in SOCIAL I that identified whether individuals were non-Hispanic White, non-Hispanic Black, Hispanic, Asian, or other (eg, Native Hawaiian or Pacific Islander, Middle Eastern or North African, or multiracial but not Hispanic).

#### Social Media

Individuals who were Twitter users (ie, all individuals in SOCIAL I and some in SOCIAL II) were queried about how much time they spent on Twitter (less than once every few weeks; every few weeks; a few days (more like 1-2) a week; a few days (3-5) a week; about once a day; or several times a day). This item was selected on the basis that it was used by the Pew Research Center in a previous study on social media use in the United States. In addition, all individuals were queried about their use of Twitter as well as other social media platforms on a binary scale (ie, user vs nonuser of that platform). We asked for a Twitter handle for all individuals in the study who identified that they were Twitter users. Individuals could choose to enter a valid Twitter handle or to enter text to bypass the question (eg “I don’t want to give my Twitter handle”).

#### Mental Health

We compiled a battery of self-report disorder screening questionnaires for psychopathology (Table 1). These measures were chosen because (1) they measure symptoms that are relatively common (eg, depression) or relatively uncommon but highly impairing (eg, drug use), (2) they are indicators of some of the major domains of psychopathology as per contemporary nosologies (eg, the study by Kotov et al [2]), (3) they were freely available, and (4) they are widely used. Most of the measures we used were the Diagnostic and Statistical Manual of Mental Disorders (DSM) severity measures recommended by the American Psychiatric Association (eg, social anxiety, panic, worry, and substance use) or were measures that were eventually adapted into the *DSM* severity measures (ie, Patient Health Questionnaire (PHQ)-9 and PHQ-15 for depression and somatic symptoms, respectively). Given that all these measures have different response types and number of items, and accordingly different ranges, we standardized them all as percentage of maximum point (POMP) scores [22]. The POMP scores are defined as follows: POMP = ((observed score – minimum possible) / (maximum possible – minimum possible)) × 100. This represents the percentage of a measure’s total that a specific score represents. For example, for the PHQ-9, with its score range of 0 to 27, a score of 0 is 0% of the POMP, 14 is 51.85%, and 27 is 100%. In addition to characterizing the symptoms of psychopathology that individuals currently experienced, we also asked them about whether they were aware of having received a medical diagnosis of the following mental disorders: depression, social anxiety, generalized anxiety, specific phobia, panic disorder, agoraphobia, posttraumatic stress disorder, somatic symptom disorder (or “chronic pain”), insomnia, alcohol use, drug use, or bipolar disorder (I or II). Individuals were allowed to answer “yes,” “no,” “no, but I should be,” or “I don’t know.” In this study, we differentiated between individuals who were sure they had a diagnosis (ie, those answering “yes”) and all others.

We conducted preliminary analyses to describe the samples, including the ranges represented in the different variables. The results of these analyses suggested that individuals gave relatively high ratings of self-reported manic symptoms, a problem that has been previously reported in the literature assessing hypomanic symptoms via self-report. Zimmerman [23] suggested that screening for bipolar disorder should be accompanied by a subsequent evaluation by a clinician. Similarly, individuals endorsed relatively few agoraphobic symptoms that were highly correlated with other internalizing symptoms. Considering these factors, we removed the mania rating scale, the Altman Self-Rating Mania Scale as well as the DSM Severity Scale For Agoraphobia from SOCIAL II leaving only a subsample of individuals with ratings on these scales (n=665).

### Twitter Status

All individuals who reported that they were Twitter users were asked to provide their Twitter handles, which identify the individual on Twitter. The Twitter application programming interface, a free and public interface provided by Twitter, provides access to an individual’s past tweets (timelines) via their individual handle (provided the tweets were public). Hence, for individuals who provided a Twitter handle, we retrieved individual timelines (a time-sorted record of their past tweets). We assessed whether the corresponding Twitter accounts were valid and belonged to real users using the Botometer application programming interface, an algorithm that uses machine learning to predict whether a given account belongs to a *bot* from its web-based behavior and content (eg, frequency of posting, specific content features, evidence that they have purchased followers, whether the account self-declares as being a bot, or whether the account has been declared a bot by others). As per recommendations of the Botometer developers, we explored the distribution of bot scores and created a cutoff of 0.42 to classify individuals as *bot-like* or *valid* users.

Individuals were classified as providing *invalid* handles if they refused to provide their handle, answered the question about handles with a response that was not a syntactically valid Twitter account (eg, “I don’t want to give you this information”), or if Botometer failed to access the Twitter account. In addition to these 3 groups (ie, *invalid, bot-like*, and *valid*), in SOCIAL II, we included individuals who denied being Twitter users (*not a Twitter user*). We focused on the differences between these 3-4 subgroups using Twitter users who did not provide handles or who provided handles that were not syntactically valid account names (ie, the *invalid* handle group).

### Analytic Plan

All analyses were conducted using the R programming language (version 4.1.2) [24] in R Studio [25]. Given that we have collected samples that differ substantially in demographic characteristics, we report all analyses according to the study cohort (ie, first in SOCIAL I and then in SOCIAL II). For continuous variables, we provide descriptive statistics in the form of means, SDs, medians, and IQR values. For categorical variables, we present frequencies and percentages.

To assess statistically significant differences between the sociodemographic factors, social media use, and mental health variables, we tested the association of each of these variables (eg, age, frequency of Twitter use, and depression) with group membership (ie, no handle, bot-like, valid, and no Twitter [on SOCIAL II]). For continuous variables, we reported the *P* values from a Kruskal-Wallis rank-sum test. For categorical variables, we reported the *P* values from a chi-square test to assess whether Twitter group membership is significantly related to specific baseline characteristics (eg, race and gender identity) or the *P* values from Fisher exact test when a cell size is <5. To characterize the magnitude of these associations (ie, the strength of the effect beyond its statistical significance), for binary variables, we report odds ratios (ORs) with 95% CIs when using individuals who provided invalid user names as the reference group. For nominal variables (eg, gender as male, female, or nonbinary), we report a Cramer V. For continuous variables, we report the standardized β values and 95% CIs, representing the differences in SD units of each variable in question.

### Ethics Approval

Both studies were approved by the Indiana University Institutional Review Board (2002549202 and 2005948214).

## Results

### SOCIAL I

#### Demographic Characteristics

In SOCIAL I (N=1123), the average participant was in their mid-30s, although there was variability in the ages represented (Table 2). Approximately half of the individuals (580/1123, 51.65%) provided valid Twitter handles. For the remainder (ie, the 543/1123, 48.35% who did not provide valid Twitter handles), most were individuals who provided invalid Twitter handles (353/1123, 31.43%) with only 16.92% (190/1123) of people providing Twitter handles that were deemed to be *bot-like*. Most of those who used Twitter reported using the platform at least “several times a day.” Individuals were approximately split along the political spectrum and there appeared to be variability in sexual orientation, gender identity, and socioeconomic status. Hispanic and Asian individuals appeared to be underrepresented relative to the population from the United States.

There were various statistically significant demographic differences among individuals in SOCIAL I based on Twitter status (Table 2). In general, we focused on differences relevant to the individuals who provided valid Twitter handles versus those who refused to provide a handle or provided an invalid one (eg, we ignored differences between people who provided invalid handles vs bot-like handles). Compared with Twitter users who provided invalid handles, Twitter users who provided valid handles were more liberal (β=–0.14, 95% CI –0.20 to –0.07), used Twitter less (OR 0.73, 95% CI 0.56-0.95), and reported lower incomes (OR 0.58, 95% CI 0.46-0.74). In addition, compared with Twitter users who provided invalid handles, Twitter users who provided valid handles were relatively more likely to identify as genderqueer, nonbinary, or otherwise unwilling to use male or female designation than to identify as male (Cramer V=0.14, 95% CI 0.11-0.19) and were relatively more likely to identify as gay, lesbian, or bisexual than as heterosexual (OR 1.59, 95% CI 1.12-2.28).

**Table 2:** Sociodemographic characteristics of web-based panel respondents to the Studies of Online Cohorts for Internalizing Symptoms and Language (SOCIAL I), overall and by Twitter status (N=1123).

Characteristic		Twitter user, bot-like handle (n=190^a^)	Twitter user, invalid handle (n=353^b^)	Twitter user, valid handle (n=580^c^)	*P* value^d^	
**Age (years)**						<.001
	Mean (SD)	38.41 (12.86)	34.20 (11.80)	33.75 (13.17)		
	Median (IQR)	36.00 (29.00-45.00)	35.00 (24.00-40.00)	31.00 (22.00-41.00)		
	Unknown, n (%)	3 (1.58)	18 (5.10)	29 (5.00)		
**Political orientation, rating (1-10; 1=extremely liberal, 10=extremely conservative)**						<.001
	Mean (SD)	5.12 (2.58)	5.31 (2.57)	4.62 (2.52)		
	Median (IQR)	5.00 (3.00-7.00)	5.00 (3.00-7.00)	5.00 (2.00-7.00)		
	Unknown, n (%)	2 (1.05)	3 (0.89)	7 (1.21)		
**Time spent on Twitter, n (%)**						.17
	Less than every few weeks	5 (2.63)	6 (1.70)	13 (2.2)		
	Every few weeks	10 (5.26)	14 (3.97)	21 (3.6)		
	A few days (eg, 1-2 days) a week	5 (2.63)	15 (4.25)	33 (5.7)		
	A few days (eg, 3-5 days) a week	16 (8.42)	31 (8.78)	77 (13)		
	About once a day	27 (14.21)	56 (15.86)	101 (17)		
	Several times a day	127 (66.84)	231 (65.44)	335 (58)		
**Race** **and** **ethnicity, n (%)**						.13
	Non-Hispanic White	148 (77.89)	262 (74.43)	416 (72)		
	Non-Hispanic Black	22 (11.58)	39 (11.08)	67 (12)		
	Hispanic	6 (3.16)	26 (7.39)	55 (9.5)		
	Asian	13 (6.84)	17 (4.83)	32 (5.5)		
	Others	1 (0.53)	8 (2.27)	10 (1.7)		
	Unknown	0 (0)	1 (0.28)	0 (0)		
**Gender, n (%)**						<.001
	Woman	80 (42.11)	163 (46.18)	80 (42.11)		
	Genderqueer or nonbinary	2 (1.05)	3 (0.85)	2 (1.05)		
	Man	108 (56.84)	187 (52.97)	108 (56.84)		
**Sexual orientation, n (%)**						.005
	Heterosexual	165 (86.84)	301 (85.27)	165 (86.84)		
	LGB^e^	25 (13.16)	52 (14.73)	25 (13.16)		
**Yearly income (US $), n (%)**						<.001
	<10,000	8 (4.21)	29 (8.22)	8 (4.21)		
	10,000-19,999	15 (7.89)	21 (5.95)	15 (7.89)		
	20,000-29,999	23 (12.11)	35 (9.92)	23 (12.11)		
	30,000-39,999	21 (11.05)	35 (9.92)	21 (11.05)		
	40,000-49,999	9 (4.74)	19 (5.38)	9 (4.74)		
	50,000-59,999	7 (3.68)	25 (7.08)	7 (3.68)		
	60,000-69,999	9 (4.74)	12 (3.40)	9 (4.74)		
	70,000-79,999	15 (7.89)	22 (6.23)	15 (7.89)		
	80,000-89,999	6 (3.16)	9 (2.55)	6 (3.16)		
	90,000-99,999	12 (6.32)	18 (5.10)	12 (6.32)		
	100,000-149,999	34 (17.89)	71 (20.11)	34 (17.89)		
	≥150,000	31 (16.32)	57 (16.15)	31 (16.32)		

^a^Bot-like handle: individual reported being a Twitter user and provided a Twitter handle, but it had a Botometer score >0.42.

^b^Invalid handle: individual reported being a Twitter user but did not provide their Twitter handle or provided a handle that was invalid.

^c^Valid handle: individual reported being a Twitter user and provided a Twitter handle, and it had a Botometer score ≤0.42.

^d^Kruskal-Wallis rank-sum test; Fisher exact test for count data with simulated *P* value (based on 2000 replicates); Pearson chi-square test.

^e^LGB: lesbian, gay, bisexual (or other nonheterosexual sexual orientation).

#### Social Media Use

In SOCIAL I, all individuals were recruited to be Twitter users. Other social media platforms used by most of the sample were Facebook, Instagram, and YouTube (Table 3). There appeared to be several statistically significant differences in social media use by Twitter status, but these effects were mostly attributable to bot-like users (eg, bot-like users were more likely to report being on LINE than valid users). Individuals who provided valid Twitter handles were more likely to report that they used Tumblr (OR 1.48, 95% CI 1.07-2.05) and Pinterest than individuals who provided invalid Twitter handles (OR 1.79, 95% CI 1.37-2.34).

**Table 3:** Social media platforms used by respondents to the Studies of Online Cohorts for Internalizing Symptoms and Language (SOCIAL), by cohort (SOCIAL I and SOCIAL II) and Twitter status.

Social media platforms	Cohort									
	SOCIAL I (N=1123)					SOCIAL II (N=1988)				
	Twitter user, bot-like handle (n=190)^a^, n (%)	Twitter user, invalid handle (n=353)^b^, n (%)	Twitter user, valid handle (n=580)^c^, n (%)	*P* value^d^	Twitter user, bot-like handle (n=229), n (%)		Not a Twitter user (n=760), n (%)	Twitter user, invalid handle (n=515), n (%)	Twitter user, valid handle (n=484), n (%)	*P* value^e^
Twitter	190 (100)	353 (100)	580 (100)	—^f^	229 (100)		0 (0)	515 (100)	484 (100)	<.001
Facebook	170 (89.47)	311 (88.1)	523 (90.17)	.61	198 (86.46)		427 (56.18)	421 (81.75)	401 (82.85)	<.001
Instagram	158 (83.16)	321 (90.93)	522 (90.00)	.01	227 (99.13)		693 (91.18)	503 (97.67)	478 (98.76)	<.001
Snapchat	103 (54.21)	220 (62.32)	361 (62.24)	.12	224 (97.82)		684 (90.00)	499 (96.89)	476 (98.35)	<.001
Tumblr	47 (24.74)	68 (19.26)	151 (26.03)	.06	26 (11.35)		40 (5.26)	64 (12.43)	60 (12.4)	<.001
YouTube	164 (86.32)	291 (82.44)	486 (83.79)	.5	198 (86.46)		534 (70.26)	418 (81.17)	380 (78.51)	<.001
TikTok	75 (39.47)	178 (50.42)	267 (46.03)	.05	200 (87.34)		516 (67.89)	425 (82.52)	424 (87.6)	<.001
Reddit	61 (32.11)	98 (27.76)	176 (30.34)	.53	48 (20.96)		98 (12.89)	111 (21.55)	91 (18.8)	<.001
4Chan	12 (6.32)	11 (3.12)	9 (1.55)	.003	1 (0.44)		1 (0.13)	0 (0)	2 (0.41)	.32
Pinterest	98 (51.58)	154 (43.63)	337 (58.1)	<.001	153 (66.81)		389 (51.18)	332 (64.47)	344 (71.07)	<.001
Twitch	51 (26.84)	69 (19.55)	119 (20.52)	.11	40 (17.47)		65 (8.55)	81 (15.73)	84 (17.36)	<.001
LINE	22 (11.58)	24 (6.8)	34 (5.86)	.03	6 (2.62)		14 (1.84)	5 (0.97)	6 (1.24)	.30
Other	8 (4.21)	9 (2.55)	18 (3.1)	.57	10 (4.37)		27 (3.55)	22 (4.27)	15 (3.1)	.73

^a^Bot-like: individual reported being a Twitter user and provided a Twitter handle, but it had a Botometer score>0.42.

^b^Invalid handle: individual reported being a Twitter user but did not provide their Twitter handle or provided a handle that was invalid.

^c^Valid handle: Individual reported being a Twitter user and provided a Twitter handle with a Botometer score ≤0.42.

^d^Pearson chi-square test.

^e^Pearson chi-square test; Fisher exact test for count data with simulated *P* value (based on 2000 replicates).

^f^Not available.

#### Mental Health

**Figure 1:**
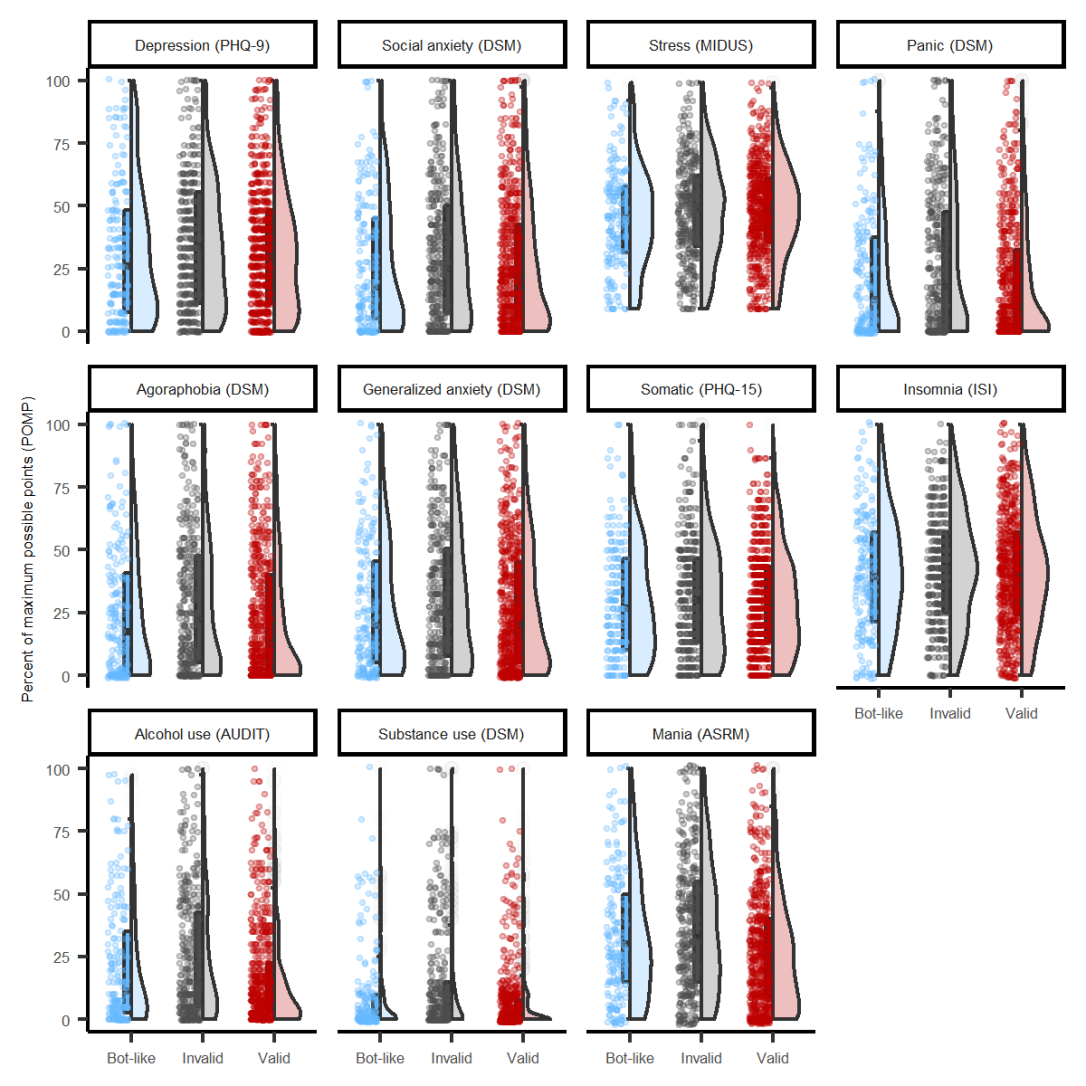
Differences in self-reported symptoms of psychopathology for 1123 individuals in the Studies of Online Cohorts for Internalizing Symptoms and Language (SOCIAL) I. ASRM: Altman Self-Rating Mania; AUDIT: Alcohol Use Disorders Identification Test; DSM: Diagnostic and Statistical Manual of Mental Disorders; ISI: Insomnia Severity Index; MIDUS: Midlife in the United States self-reported measure of perceived stress; PHQ: Patient Health Questionnaire.

The POMP scores for the various measures of psychopathology as well as the reported diagnoses are presented in Figure 1 and Table 4. Stress and insomnia were the most commonly endorsed symptoms. Major depression and generalized and social anxiety were the most commonly reported clinical diagnoses. There were a few statistically significant differences between the groups in clinical symptoms or diagnoses. The differences we did find were very small. For example, the largest difference between the groups was in self-reported manic symptoms and suggested individuals who provided valid Twitter handles had lower symptoms of hypomania than individuals who did not provide Twitter handles, although this difference was small by conventional standards (β=–0.22, 95% CI –0.28 to –0.15. The next highest difference between the groups was in self-reported issues with alcohol and suggested that individuals who provided valid Twitter handles had lower alcohol use symptoms than individuals who did not provide Twitter handles, although this difference was small (β=–0.19, 95% CI –0.25 to –0.12). Compared with individuals who provided invalid handles, individuals who provided valid handles were less likely to report relatively rare diagnoses, such as somatic symptom disorder (OR 0.38, 95% CI 0.21-0.68) and drug use disorder (OR 0.60, 95% CI 0.39-0.92)

**Table 4:** Median (IQR) of symptom severity, as percentage of maximum points (0-100) and self-reported diagnoses of psychopathology in web-based respondents to the Studies of Online Cohorts for Internalizing Symptoms and Language (SOCIAL I), by Twitter status (N=1123).

Variable			Twitter user, bot-like handle (n=190^a^)		Twitter user, invalid handle (n=353^b^)		Twitter user, valid handle (n=580^c^)		*P* value^d^	
**Social anxiety (DSM^e^)**										.02
	Median (IQR)	17.50 (5.00-45.00)		25.00 (7.50-50.00)		20.00 (5.00-42.50)				
	Unknown, n (%)	2 (1.05)		1 (0.28)		0 (0)				
**Stress (MIDUS^f^)**										.17
	Median (IQR)	45.00 (31.50-57.50)		49.00 (34.00-62.00)		48.00 (36.00-61.00)				
	Unknown (n)	3 (1.58)		0 (0)		0 (0)				
**Depression (PHQ^g^-9)**										.21
	Median (IQR)	25.93 (7.41-48.15)		33.33 (11.11-55.56)		29.63 (11.11-48.15)				
	Unknown, n (%)	2 (1.05)		1 (0.28)		0 (0)				
**Panic (DSM)**										<.001
	Median (IQR)	12.50 (0.00-37.50)		17.50 (0.00-47.50)		7.50 (0.00-32.50)				
	Unknown, n (%)	2 (1.05)		2 (0.57)		0 (0)				
**Agoraphobia (DSM)**										<.001
	Median (IQR)	17.50 (0.00-40.62)		22.50 (5.00-47.50)		12.50 (0.00-40.00)				
	Unknown, n (%)	2 (1.05)		1 (0.28)		1 (0.17)				
**Generalized anxiety (DSM)**										.04
	Median (IQR)	20.00 (5.00-45.62)		25.00 (7.50-50.62)		20.00 (5.00-45.00)				
	Unknown, n (%)	2 (1.05)		1 (0.28)		1 (0.17)				
**Somatic (PHQ-15)**										.75
	Median (IQR)	26.67 (10.00-46.67)		30.00 (13.33-46.67)		26.67 (13.33-43.33)				
	Unknown, n (%)	5 (2.63)		7 (1.98)		2 (0.34)				
**Insomnia (ISI^h^)**										.38
	Median (IQR)	39.29 (21.43-57.14)		42.86 (25.00-57.14)		39.29 (24.11-57.14)				
	Unknown, n (%)	39.29 (21.43-57.14)		42.86 (25.00-57.14)		39.29 (24.11-57.14)				
Alcohol use (AUDIT^i^), median (IQR)			11.25 (2.50-35.00)		15.00 (5.00-42.50)		7.50 (2.50-22.50)		<.001	
**Substance use (DSM)**										<.001
	Median (IQR)	2.50 (0.00-10.00)		5.00 (0.00-15.00)		0.00 (0.00-7.50)				
	Unknown, n (%)	1 (0.52)		1 (0.28)		4 (0.68)				
**Hypomania (ASRM^j^ scale)**										<.001
	Median (IQR)	30.00 (15.00-50.00)		32.50 (15.00-55.00)		25.00 (8.75-40.00)				
	Unknown, n (%)	3 (1.58)		1 (0.28)		0 (0)				
**Insomnia Dx^k^, n (%)**										.45
	Yes	48 (25.53)		89 (25.36)		127 (22.2)				
	Unknown	2 (1.05)		2 (0.57)		8 (1.38)				
**Somatic symptom Dx, n (%)**										.003
	Yes	8 (4.26)		30 (8.62)		20 (3.50)				
	Unknown	2 (1.05)		5 (1.41)		8 (1.38)				
**Major depression Dx, n (%)**										.57
	Yes	73 (38.62)		147 (42.12)		247 (43.03)				
	Unknown	1 (0.52)		4 (1.13)		6 (1.03)				
**Specific phobia Dx, n (%)**										.10
	Yes	28 (14.89)		62 (17.77)		72 (12.59)				
	Unknown	2 (1.05)		4 (1.13)		8 (1.38)				
**Social anxiety Dx, n (%)**										.10
	Yes	37 (19.68)		96 (27.67)		153 (26.7)				
	Unknown	2 (1.05)		6 (1.70)		7 (1.21)				
**Panic Dx, n (%)**										.79
	Yes	35 (18.82)		68 (19.60)		102 (17.8)				
	Unknown	4 (2.10)		6 (1.70)		7 (1.21)				
**Posttraumatic stress Dx, n (%)**										.32
	Yes	33 (17.74)		46 (13.26)		93 (16.29)				
	Unknown	4 (2.10)		6 (1.70)		9 (1.55)				
**Generalized anxiety Dx, n (%)**										.08
	Yes	50 (26.74)		92 (26.44)		188 (32.75)				
	Unknown	3 (1.58)		5 (1.41)		6 (1.03)				
**Agoraphobia Dx, n (%)**										.25
	Yes	9 (4.81)		26 (7.51)		29 (5.07)				
	Unknown	3 (1.58)		7 (1.98)		8 (1.38)				
**Alcohol use Dx, n (%)**										.11
	Yes	23 (12.30)		42 (12.07)		48 (8.35)				
	Unknown	3 (1.58)		5 (1.41)		5 (0.86)				
**Substance use Dx, n (%)**										.048
	Yes	23 (12.37)		46 (13.22)		48 (8.39)				
	Unknown	4 (2.10)		5 (1.41)		8 (1.38)				
**Bipolar Dx, n (%)**										.68
	Yes	23 (12.23)		40 (11.49)		58 (10.18)				
	Unknown	2 (1.05)		5 (1.41)		10 (1.72)				

^a^Bot-like: individual reported being a Twitter user and provided a Twitter handle, but it had a Botometer score >0.42.

^b^Invalid handle: individual reported being a Twitter user but did not provide their Twitter handle or provided a handle that was invalid.

^c^Valid handle: individual reported being a Twitter user and provided a Twitter handle, and it had a Botometer score ≤0.42.

^d^Kruskal-Wallis rank-sum test; Pearson chi-square test.

^e^DSM: Diagnostic and Statistical Manual of Mental Disorders.

^f^MIDUS: Midlife in the United States self-reported measure of perceived stress.

^g^PHQ: Patient Health Questionnaire.

^h^ISI: Insomnia Severity Index.

^i^AUDIT: Alcohol Use Disorders Identification Test.

^j^ASRM: Altman Self-Rating Mania.

^k^Dx: diagnosis.

### Studies of Online Cohorts for Internalizing Symptoms and Language II

#### Demographic Characteristics

In SOCIAL II (N=1988), age was more restricted to the range 18 to 22 years, as would be expected of undergraduate students (mean 19.07, SD 2.91 years; Table 5). The sample was primarily female (Table 5), as is typical of psychology students, and primarily White and Asian, which is consistent with the demographic characteristics of the institution. A total of 32.22% (760/1128) of participants denied being Twitter users. Of the Twitter users (1228/1988, 61.77% of the whole sample), most either refused to give handles (515/1228, 41.94%) or gave valid handles (484/1228, 39.41%), with a minority providing handles that were deemed to be bot-like (229/1228, 18.65%). Of those who reported being Twitter users, approximately half of the individuals used Twitter “about once a day.” Approximately half of all the students reported that their parents made ≥US $100,000 and the others were distributed relatively uniformly across the income categories. Politically, they reported aligning with the politics center of the liberal-conservative continuum.

There were 3 statistically significant differences between individuals based on their Twitter user status, of which 2 involved the individuals with valid Twitter user names. First, individuals who provided valid Twitter handles used Twitter more frequently than individuals who were Twitter users but did not provide their handles or provided invalid handles (OR 2.48, 95% CI 1.98-3.13). Second, there were differences in reported race and ethnicity by Twitter user status (Cramer V=0.03, 95% CI 0.02-0.07). Specifically, individuals who provided valid Twitter handles were less likely to be Hispanic than individuals who were Twitter users but did not provide their handles (OR 0.40, 95% CI 0.21-0.77).

**Table 5:** Sociodemographic characteristics of young adult respondents to the Studies of Online Cohorts for Internalizing Symptoms and Language (SOCIAL II), overall and by Twitter status (N=1988).

Characteristic			Twitter user, bot-like handle (n=229)^a^		Not a Twitter user (n=760)		Twitter user, invalid handle (n=515)^b^		Twitter user, valid handle (n=484)^c^		*P* value^d^	
**Age (years)**												.02
	Mean (SD)	18.91 (1.55)		19.11 (4.09)		19.13 (2.44)		19.03 (1.12)				
	Median (IQR)	19.00 (18.00-19.00)		19.00 (18.00-19.00)		19.00 (18.00-19.00)		19.00 (18.00-20.00)				
	Unknown, n (%)	15 (6.55)		50 (6.58)		41 (7.96)		25 (5.17)				
**Conservatives, rating (1-10; 1=extremely liberal, 10=extremely conservative)**												.22
	Mean (SD)	4.12 (2.07)		4.18 (1.94)		4.03 (2.07)		3.96 (2.09)				
	Median (IQR)	4.00 (2.00-5.25)		4.00 (3.00-5.00)		4.00 (2.00-5.00)		4.00 (2.00-6.00)				
	Unknown, n (%)	5 (2.18)		27 (3.55)		30 (5.82)		8 (1.65)				
**Time spent on Twitter, n (%)**												<.001
	Less than every few weeks	64 (27.95)		N/A^e^		115 (23.23)		29 (5.99)				
	Every few weeks	46 (20.09)		N/A		85 (17.17)		43 (8.88)				
	A few days (eg, 1-2 days) a week	33 (14.41)		N/A		48 (9.70)		67 (13.84)				
	A few days (eg, 3-5 days) a week	19 (8.30)		N/A		30 (6.06)		39 (8.06)				
	About once a day	38 (16.59)		N/A		86 (17.37)		112 (23.14)				
	Several times a day	29 (12.66)		N/A		131 (26.46)		194 (40.08)				
	Unknown	0 (0)		760 (100)		20 (3.88)		0 (0.00)				
**Race** **and** **ethnicity, n (%)**												<.001
	Non-Hispanic White	167 (73.57)		519 (68.92)		357 (72.71)		376 (78.50)				
	Asian	27 (11.89)		116 (15.41)		44 (8.96)		34 (7.10)				
	Non-Hispanic Black	18 (7.93)		50 (6.64)		44 (8.96)		45 (9.39)				
	Hispanic	13 (5.73)		44 (5.84)		33 (6.72)		14 (2.92)				
	Others	2 (0.88)		24 (3.19)		13 (2.65)		10 (2.09)				
	Unknown	2 (0.87)		7 (0.92)		24 (4.66)		5 (1.03)				
**Gender, n (%)**												.75
	Woman	179 (78.85)		573 (76.10)		373 (75.97)		368 (76.83)				
	Genderqueer or nonbinary	1 (0.44)		8 (1.06)		9 (1.83)		4 (0.84)				
	Man	47 (20.70)		172 (22.84)		109 (22.20)		107 (22.34)				
	Unknown	2 (0.87)		7 (0.92)		24 (4.66)		5 (1.03)				
**Sexual orientation, n (%)**												.16
	Heterosexual	200 (88.11)		638 (84.73)		401 (81.67)		401 (83.72)				
	LGB^f^	27 (11.89)		115 (15.27)		90 (18.33)		78 (16.28)				
	Unknown	2 (0.87)		7 (0.92)		24 (4.66)		5 (1.03)				
**Yearly income (US $), n (%)**												.99
	<10,000	13 (5.75)		37 (5.00)		34 (6.98)		20 (4.22)				
	10,000-19,999	5 (2.21)		23 (3.11)		16 (3.29)		18 (3.80)				
	20,000-29,999	12 (5.31)		32 (4.32)		23 (4.72)		19 (4.01)				
	30,000-39,999	9 (3.98)		39 (5.27)		20 (4.11)		25 (5.27)				
	40,000-49,999	9 (3.98)		37 (5.00)		20 (4.11)		22 (4.64)				
	50,000-59,999	13 (5.75)		37 (5.00)		21 (4.31)		22 (4.64)				
	60,000-69,999	9 (3.98)		36 (4.86)		26 (5.34)		26 (5.49)				
	70,000-79,999	13 (5.75)		38 (5.14)		21 (4.31)		24 (5.06)				
	80,000-89,999	15 (6.64)		39 (5.27)		23 (4.72)		29 (6.12)				
	90,000-99,999	13 (5.75)		51 (6.89)		40 (8.21)		37 (7.81)				
	100,000-149,999	41 (18.14)		140 (18.92)		104 (21.36)		103 (21.73)				
	≥150,000	74 (32.74)		231 (31.22)		139 (28.54)		129 (27.22)				
	Unknown	3 (1.31)		20 (2.63)		28 (5.44)		10 (2.07)				

^a^Bot-like: individual reported being a Twitter user and provided a Twitter handle, but it had a Botometer score >0.42.

^b^Invalid handle: individual reported being a Twitter user but did not provide their Twitter handle or provided a handle that was invalid.

^c^Valid handle: individual reported being a Twitter user and provided a Twitter handle and it had a Botometer score ≤0.42.

^d^Kruskal-Wallis rank-sum test; Pearson chi-square test; Fisher exact test for count data with simulated *P* value (based on 2000 replicates).

^e^N/A: not applicable.

^f^LGB: lesbian, gay, bisexual (or other nonheterosexual sexual orientation).

#### Social Media

In SOCIAL II, Instagram and Snapchat were the most popular platforms and were used by almost all individuals (Table 3). Twitter, Facebook, YouTube, and TikTok were also quite popular, being used by 61.77% (1228/1988) to 78.72% (1530/1988) of the sample. A number of differences emerged with respect to which social media platforms the participants used. However, most of these differences indicated that individuals who denied being Twitter users were also less likely to use other platforms. The 2 exceptions were that the individuals in SOCIAL II who used Twitter and provided valid handles were more likely to also use TikTok (OR 1.50, 95% CI 1.05-2.14) and Pinterest (OR 1.35, 95% CI 1.04-1.77) than the individuals who refused to provide handles or provided invalid handles.

#### Mental Health

**Figure 2:**
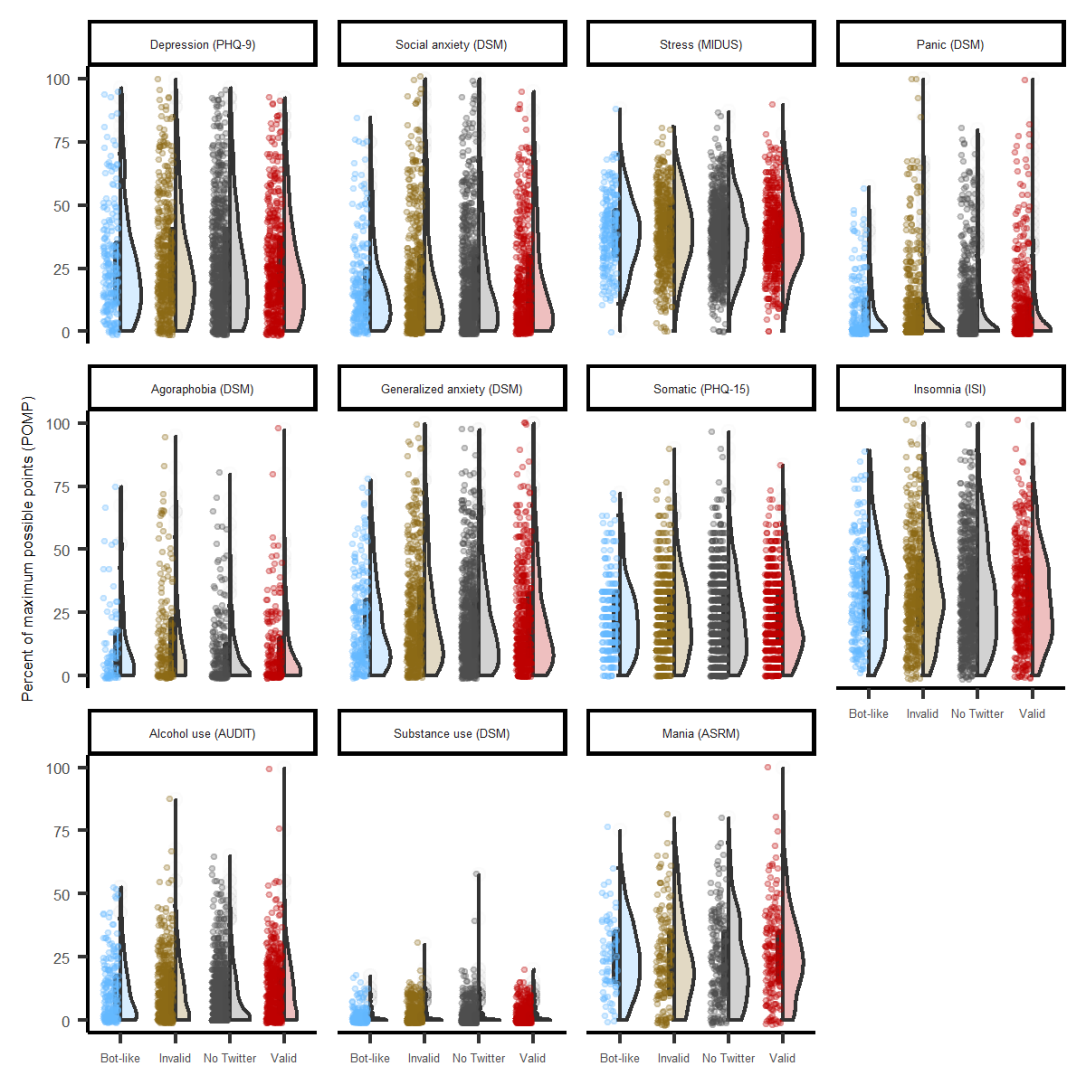
Symptoms and self-reported diagnoses of psychopathology in 1988 college students responding to the Studies of Online Cohorts for Internalizing Symptoms and Language (SOCIAL II). ASRM: Altman Self-Rating Mania; AUDIT: Alcohol Use Disorders Identification Test; DSM: Diagnostic and Statistical Manual of Mental Disorders; ISI: Insomnia Severity Index; MIDUS: Midlife in the United States self-reported measure of perceived stress; PHQ: Patient Health Questionnaire.

The POMP scores for the various measures of psychopathology as well as the reported diagnoses are presented in Figure 2 and Table 6. Similar to SOCIAL I, in SOCIAL II, stress and insomnia were the most commonly endorsed symptoms, and major depression and generalized and social anxiety were the most commonly reported clinical diagnoses. Relative to SOCIAL I (Table 4), there were even fewer statistically significant differences between the groups in clinical symptoms or diagnoses. The largest difference between individuals who provided valid (vs invalid) handles was in agoraphobic symptoms, and it was relatively small in magnitude (β=–0.13, 95% CI 0.03-0.15).

**Table 6:** Median (IQR) of symptom severity, as percentage of maximum points (0-100) and self-reported diagnoses of psychopathology in web-based respondents in the Studies of Online Cohorts for Internalizing Symptoms and Language (SOCIAL I), by Twitter status (N=1123).

Variable		Twitter user, bot-like (n=229)^a^	Not a Twitter user (n=760)	Twitter user, invalid handle (n=515)^b^	Twitter user, valid handle (n=484)^c^	*P* value^d^	
**Social anxiety (DSM^e^)**							.63
	Median (IQR)	15.00 (5.00-25.00)	12.50 (5.00-30.00)	12.50 (5.00-30.00)	12.50 (2.50-30.00)		
	Unknown, n (%)	2 (0.87)	5 (0.66)	22 (4.27)	2 (0.41)		
**Stress (MIDUS^f^)**							.07
	Median (IQR)	39.00 (30.00-48.00)	38.00 (27.00-48.00)	39.00 (29.00-50.00)	37.00 (28.00-47.00)		
	Unknown, n (%)	0 (0)	4 (0.53)	21 (4.08)	0 (0)		
**Depression (PHQ^g^-9)**							.33
	Median (IQR)	22.22 (11.11-35.19)	22.22 (11.11-40.74)	22.22 (11.11-40.74)	22.22 (11.11-37.04)		
	Unknown, n (%)	2 (0.87)	4 (0.53)	22 (4.27)	1 (0.21)		
**Panic (DSM)**							.91
	Median (IQR)	2.50 (0.00-12.50)	2.50 (0.00-12.50)	2.50 (0.00-12.50)	2.50 (0.00-12.50)		
	Unknown, n (%)	2 (0.87)	5 (0.66)	22 (4.27)	2 (0.41)		
**Agoraphobia (DSM)**							.006
	Median (IQR)	5.00 (0.00-17.50)	5.00 (0.00-12.50)	7.50 (0.00-22.50)	5.00 (0.00-15.00)		
	Unknown, n (%)	149 (65.06)	533 (70.13)	343 (66.60)	298 (61.57)		
**Generalized anxiety (DSM)**							.40
	Median (IQR)	15.00 (7.50-30.00)	17.50 (7.50-30.00)	17.50 (7.50-35.00)	15.00 (7.50-32.50)		
	Unknown, n (%)	2 (0.87)	7 (0.92)	23 (4.47)	2 (0.41)		
**Somatic (PHQ-15)**							.70
	Median (IQR)	20.00 (10.00-33.33)	20.00 (10.00-33.33)	23.33 (13.33-33.33)	20.00 (13.33-33.33)		
	Unknown, n (%)	2 (0.87)	8 (1.05)	25 (4.85)	2 (0.41)		
**Insomnia (ISI^h^)**							.22
	Median (IQR)	32.14 (17.86-46.43)	28.57 (17.86-46.43)	32.14 (17.86-46.43)	28.57 (17.86-42.86)		
	Unknown, n (%)	1 (0.44)	4 (0.53)	21 (4.08)	0 (0)		
**Alcohol use (AUDIT^i^)**							<.001
	Median (IQR)	7.50 (2.50-17.50)	5.00 (0.00-15.00)	10.00 (2.50-20.00)	10.00 (2.50-20.00)		
	Unknown, n (%)	2 (0.87)	8 (1.05)	25 (4.85)	3 (0.62)		
**Substance use (DSM)**							.02
	Median (IQR)	0.00 (0.00-2.50)	0.00 (0.00-2.50)	0.00 (0.00-2.50)	0.00 (0.00-2.50)		
	Unknown, n (%)	2 (0.87)	5 (0.66)	21 (4.08)	0 (0)		
**Mania or Hypomania (ASRM^j^)**							.19
	Median (IQR)	25.00 (15.00-35.00)	20.00 (10.00-35.00)	20.00 (10.00-35.00)	25.00 (15.00-35.00)		
	Unknown, n (%)	149 (65.06)	533 (70.13)	343 (66.60)	298 (61.57)		
**Insomnia Dx^k^, n (%)**							.17
	Yes	8 (3.49)	52 (6.90)	33 (6.68)	24 (4.97)		
	Unknown	0 (0)	6 (0.79)	21 (4.08)	1 (0.21)		
**Somatic symptom Dx, n (%)**							.03
	Yes	2 (0.88)	11 (1.46)	1 (0.20)	1 (0.21)		
	Unknown	1 (0.44)	6 (0.79)	21 (4.08)	1 (0.21)		
**Major depression Dx, n (%)**							.30
	Yes	50 (21.83)	183 (24.27)	136 (27.53)	129 (26.71)		
	Unknown	0 (0)	6 (0.79)	21 (4.08)	1 (0.21)		
**Specific phobia Dx, n (%)**							.58
	Yes	10 (4.39)	21 (2.79)	17 (3.44)	13 (2.69)		
	Unknown	1 (0.44)	6 (0.79)	21 (4.08)	1 (0.21)		
**Social anxiety Dx, n (%)**							.62
	Yes	32 (14.04)	87 (11.54)	68 (13.77)	62 (12.84)		
	Unknown	1 (0.44)	6 (0.79)	21 (4.08)	1 (0.21)		
**Panic Dx, n (%)**							.43
	Yes	13 (5.7)	41 (5.44)	37 (7.49)	26 (5.38)		
	Unknown	1 (0.44)	6 (0.79)	21 (4.08)	1 (0.21)		
**Posttraumatic stress Dx, n (%)**							.50
	Yes	6 (2.63)	36 (4.77)	22 (4.45)	18 (3.73)		
	Unknown	1 (0.44)	6 (0.79)	21 (4.08)	1 (0.21)		
**Generalized anxiety Dx, n (%)**							.11
	Yes	69 (30.13)	193 (25.60)	155 (31.38)	146 (30.23)		
	Unknown	0 (0)	6 (0.79)	21 (4.08)	1 (0.21)		
**Agoraphobia Dx, n (%)**							.88
	Yes	0 (0)	4 (0.53)	2 (0.40)	3 (0.62)		
	Unknown	1 (0.44)	6 (0.79)	21 (4.08)	1 (0.21)		
**Alcohol use Dx, n (%)**							.34
	Yes	0 (0)	6 (0.80)	4 (0.81)	1 (0.21)		
	Unknown	1 (0.44)	6 (0.79)	21 (4.08)	1 (0.21)		
**Substance use Dx, n (%)**							.23
	Yes	1 (0.44)	16 (2.12)	9 (1.82)	5 (1.04)		
	Unknown	1 (0.44)	6 (0.79)	21 (4.08)	1 (0.21)		
**Bipolar Dx, n (%)**							.005
	Yes	0 (0)	23 (3.05)	9 (1.82)	6 (1.24)		
	Unknown	1 (0.44)	6 (0.79)	21 (4.08)	1 (0.21)		

^a^Bot-like: individual reported being a Twitter user and provided a Twitter handle, but it had a Botometer score >0.42.

^b^Invalid handle: individual reported being a Twitter user but did not provide their Twitter handle or provided a handle that was invalid.

^c^Valid handle: individual reported being a Twitter user and provided a Twitter handle, and it had a Botometer score ≤0.42.

^d^Kruskal-Wallis rank-sum test; Pearson chi-square test; Fisher exact test for count data with simulated *P* value (based on 2000 replicates).

^e^DSM: Diagnostic and Statistical Manual of Mental Disorders.

^f^MIDUS: Midlife in the United States self-reported measure of perceived stress.

^g^PHQ: Patient Health Questionnaire.

^h^ISI: Insomnia Severity Index.

^i^AUDIT: Alcohol Use Disorders Identification Test.

^j^ASRM: Altman Self-Rating Mania.

^k^Dx: diagnosis.

## Discussion

### Principal Findings

Our results suggest that it is feasible to collect social media data from individuals who also provide information about a breadth of mental health symptoms. We found no evidence that individuals who provide valid Twitter accounts are a biased sample when compared with individuals who provide invalid handles, do not provide their handles, do not use Twitter, or are classified as bot-like. The widespread availability of social media [10] has facilitated research on large samples with longitudinal observations [26-28]. Although the nature of social media activity can be fairly simple (eg, posting short bits of text and sharing audiovisual content), researchers have made well-supported inferences from this activity about the way mood [26-28], sleep patterns [11], social relations [26], and personality [28] manifest in real-world contexts. Most of this work lacks the measurement of clinically relevant variables, such as the validated assessments of depression, anxiety, and other mental disorder symptoms that we used. For example, we conducted a study characterizing the language of individuals who self-identified as having received a clinical diagnosis of depression [15], finding that they use language that is more negative and rigid than that of a random sample [15]. Although the findings obtained using individual self-identification are interesting, they are subject to a variety of possible sample and observation biases and bear replication against validated clinical screening scales such as the ones we used in this study.

We conducted the SOCIAL I and SOCIAL II studies to triangulate data and meta-data obtained from social media with a range of validated clinical self-reports of symptoms of distress (ie, depression, stress, and generalized anxiety), fear (ie, panic and social anxiety), substance use (ie, alcohol and other drugs), somatoform problems (ie, insomnia and chronic pain), and potential thought disorder symptoms (ie, symptoms consistent with hypomania). However, a concern about studies triangulating clinical data and social media data remains that individuals who volunteer their social media accounts in such studies are not representative of individuals on social media in general [20]. In this report, we compared the baseline sociodemographic, clinical, and social media variables of individuals who were Twitter users who provided valid Twitter handles to Twitter users who provided handles associated with accounts with high bot scores, Twitter users who provided invalid account names, and, in SOCIAL II, non-Twitter users. In both cohorts, individuals who provided valid Twitter handles tended to use Twitter less than individuals who did not provide handles or who provided invalid handles, although these differences were small, and most individuals reported using Twitter “several times a day.” By and large, the differences between the groups were not statistically significant, and when they were statistically significant, they were small in magnitude. This suggests that prior work that focuses on individuals who self-disclose valid Twitter handles is generalizable, at least with regard to the demographic, clinical, and social media features measured here. We observed other demographic differences between the 2 cohorts. For example, in SOCIAL I, cisgender women were more likely to provide their handles, as were lesbian, gay, bisexual, and queer individuals (vs heterosexual individuals) and those who reported lower (vs higher) incomes. Nonetheless, in all cases in which we did detect differences, there was complete overlap in the distributions of continuous and ordinal variables, and the differences in effect sizes were relatively small in magnitude. Again, these results are encouraging regarding the generalizability of research on people who volunteer their handles to social media users more broadly, and therefore, do not support the critique that relying on a sample of users who are willing to provide their Twitter handles will lead to significant sample bias.

### Limitations and Strengths

Some limitations inherent in our data are worth considering. First, social media use, especially frequent social media use, is not a random and normally distributed variable. Evidence suggests, for example, that a small portion of users are responsible for a large number of tweets. Thus, future analyses of the SOCIAL I and II data sets and related data sets should consider the frequency of social media activity as well as the nature of that activity. In addition, the decision to enter a study focused on social media may in itself introduce a selection bias that we cannot guard against. Although our samples allow us to study mental health and social media across units of analysis (ie, self-report, text data, and meta-data), we lack more objective data including biomarkers or even observer reports of mental health symptoms. Importantly, although we did not conduct semistructured interviews about mental disorder diagnoses, the diagnosis of mental disorders is largely influenced by the severity of symptoms [29]. For many clinical problems such as depression [30], anxiety, and alcohol use [31], scores on disorder screening scales such as the ones we used are excellent predictors of diagnoses in clinical interviews.

### Future Directions

Despite the fact that social media samples are not representative of the entire population, social media users represent 20%-70% of all individuals in the United States [10], thereby providing a sample that constitutes a plurality of the entire population in the United States. In SOCIAL I, we collected a relatively heterogeneous sample of Twitter users. SOCIAL II was a more homogeneous sample, but it had the advantage of containing a subsample of individuals who did not use Twitter or were unwilling to share these data. We collected an assortment of transdiagnostic features of psychopathology representing the most common symptoms of poor mental health. These data will allow us to assess how the spectrum and range of psychopathology manifests itself in natural language and social networks.

With these data sets, we can triangulate self-reported clinical data and data collected from social media. In both samples, mental health symptoms were relatively well represented, making them good, large-scale samples for studying psychopathology. Our current analyses suggest that individuals in these data sets who volunteered to give their Twitter handle, and provided a valid handle, were not different from other individuals in terms of their demographic characteristics, social media use, and mental health. A future direction for this line of work is to use self-reported mental health to replicate findings in which mental health is inferred through social media activity. Another direction is to extend the data collection to include ecological momentary assessments to triangulate to what extent social media behavior is a valid window into individuals’ mental health.

## Abbreviations

DSM: Diagnostic and Statistical Manual of Mental Disorders
OR: odds ratio
PHQ: Patient Health Questionnaire
POMP: percentage of maximum point
SOCIAL: Studies of Online Cohorts for Internalizing Symptoms and Language
